# Obesity-related cancer and bariatric surgery: A comprehensive systematic review and meta-analysis protocol

**DOI:** 10.1371/journal.pone.0306623

**Published:** 2024-07-23

**Authors:** Isadora de Albuquerque Falcão Feitosa, Caio Cesar de Souza Castro, André Igor Nogueira de Araújo, Bárbara Scarlett Coutinho, Amália Cínthia Meneses do Rêgo, Edilmar de Moura Santos, Kleyton Santos de Medeiros, Irami Araújo-Filho

**Affiliations:** 1 Instituto de Ensino, Pesquisa e Inovação. Liga Contra o Câncer, Natal, Rio Grande do Norte, Brazil; 2 Federal University of Rio Grande do Norte, Natal, Rio Grande do Norte, Brazil; 3 Potiguar University, Natal, Rio Grande do Norte, Brazil; 4 Department of Surgery, Federal University of Rio Grande do Norte, Natal, Rio Grande do Norte, Brazil; King’s College Hospital NHS Trust: King’s College Hospital NHS Foundation Trust, UNITED KINGDOM OF GREAT BRITAIN AND NORTHERN IRELAND

## Abstract

**Introduction:**

Obesity is a silent pandemic affecting all ages and is a component of metabolic syndrome. Its treatment is conducted by lifestyle and behavioral changes, pharmacological therapy, and when correctly indicated, bariatric surgery. In recent years, the procedures for weight loss have been investigated due to their relationship with the development of many types of cancer. Although many studies have shown that bariatric surgery decreases cancer risk, other researchers observed an increase in this association. Carcinogenesis is affected by many factors, such as age, sex, type of cancer, and the bariatric surgery performed on each patient. This systematic review and meta-analysis protocol aims to clarify the association between the different modalities of bariatric surgery and the risk of cancer development in adult patients with metabolic syndrome.

**Method and analysis:**

The proposed systematic review and meta-analysis will be reported conforming to the Preferred Reporting Items for Systematic Reviews and Meta-Analyses (PRISMA-P) guidelines. This research will include observational studies (case-control and cohort studies) about patients who undergo bariatric surgery due to metabolic syndrome. Will be accepted in any language and any year. Publications without peer review will be excluded from this review. Data will be entered into the Review Manager software (RevMan5.2.3). We extracted or calculated the OR and 95% CI for dichotomous outcomes for each study. In case of heterogeneity (I^2^>50%), the random-effects model will combine the studies to calculate the OR and 95% CI.

**Ethics and dissemination:**

This study will review the published data; Thus, obtaining ethical approval is unnecessary. The findings of this systematic review will be published in a peer-reviewed journal.

**Prospero registration number:**

CRD42023432079.

## Introduction

### The problem, condition, or issue

Obesity is a progressive and deadly disease, defined by BMI ≥ 30 kg/m2 and which nowadays is reported as a silent pandemic and a global health problem. Recent studies estimated that two billion adults are obese or overweight [1].

To institute criteria for a disease, long debates between physicians, sociologists, and philosophers took place (US Department of Health and Human Services, 3^rd^ edition); upon the perception of an illness, numerous factors are relevant, such as culture, gender, historical time, and class [2].

This condition is associated with some of contemporary society’s life habits, like sleep disruption, hyper-palatable eating, high stress, indiscriminate use of medication, and a sedentary lifestyle, which formulates a non-physiological environment for the human body [3].

Obesity, besides being a disease, is also a risk factor for developing several diseases and cardiovascular, respiratory, and liver disorders. In addition, new studies show that an elevated body mass index is associated with a higher risk of some cancers. This group of malignant tumors, more prevalent among the obese population, are called obesity-related cancers [4]. They are gaining more notoriety with the increased understanding of their pathophysiological mechanisms, even though the relationship between obesity and cancer is unclear [5].

Some possible explanations are hormonal influence, chronic inflammation, or anatomical repercussions related to obesity. Studies show that obesity can cause changes in baseline levels of insulin, insulin-like growth factor-1 (IGF-1), leptin, adiponectin, steroid hormones, and some cytokines. Each of these disorders creates, in a different way, favorable conditions for oncogenesis [2].

**Fig 1:**
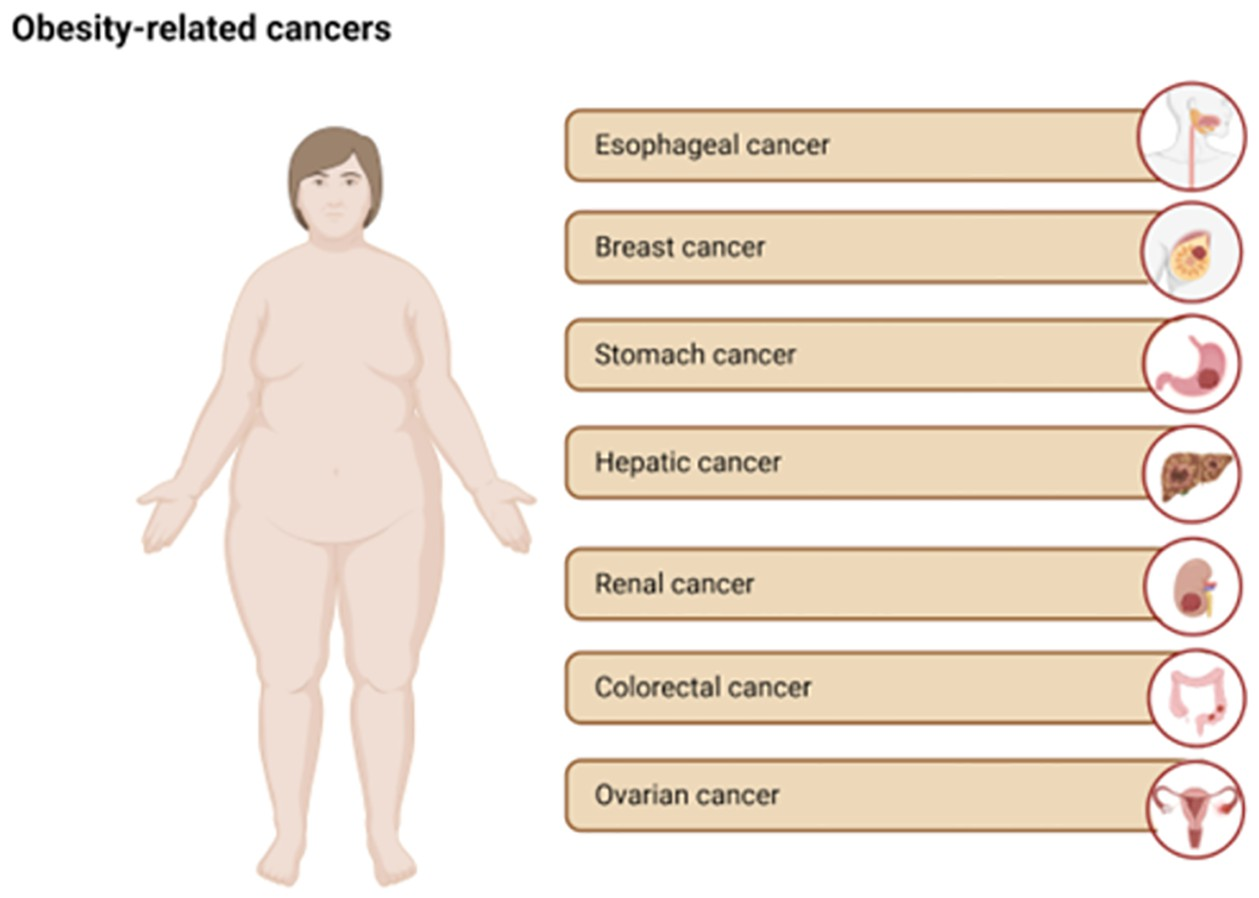
Obesity-related cancers.

Among obesity-related cancers, some tumor types stand out: hormone-related cancers (breast, endometrium, and prostate), colorectal, esophagus, and gastric cardia, since there is sufficient evidence of their relationship with human cancer [4, 5] (Fig 1).

It is also important to mention the financial impact caused by these disorders in the health systems: for example, in Brazil, in 2018, the federal cost for all types of cancer was estimated at Int$1.73 billion, of which Int$710 million came from obesity-related cancers [6].

Furthermore, there are a variety of obesity treatments available, but bariatric surgery, also known as obesity surgery, is, now, the most effective one. According to the International Federation of Surgery for Obesity and Metabolic Diseases (IFSO), the most used surgical techniques are vertical sleeve Gastrectomy Surgery (VSG) and Roux-en-Y Gastric Bypass (RYGB) [7].

### How the intervention might work

Bariatric surgical interventions interfere with weight loss through three main mechanisms: restriction of gastric volume, hormonal response, and malabsorption. Roux-en-Y gastric bypass (RYGB) combines two mechanisms: restriction and malabsorption. First, a proximal gastric pouch is created, which is then anastomosed to a Roux branch of the small intestine, resulting in gastric volume restriction. At the same time, food’s central digestion and absorption will occur in the standard channel in the presence of gastric acid, pepsin, pancreatic enzymes, and bile [7].

Sleeve Gastrectomy is a more straightforward surgical technique in which most of the stomach’s greater curvature is removed. It forms a tubular structure with reduced volumetric capacity, and the mucosal remnant has few ghrelin-producing cells, contributing to weight loss due to hormonal response [6].

The carcinogenic effect is reduced after surgery due to caloric restriction and the practice of physical exercises, benefiting metabolic mediators such as insulin, steroids, fatty hormones, inflammatory mediators, and cellular proteins [8].

From a physiological point of view, in RYGB, leptin increases, translating satiety to the hypothalamus; ghrelin reduces, inhibiting hunger in the individual; peptide 1, which resembles glucagon, increases, inhibiting the desire to ingest food [9].

SG reduces ghrelin levels, discourages appetite, and induces a positive energy balance that can lead to weight gain. At the same time, it increases GLP-1 and Peptide YY, making the individual less hungry, improving insulin resistance, and aiding glycemic control [7].

### Why it is important to do this review?

Therefore, bariatric surgery emerges as a strategy to reduce the risk of cancer in the obese population. However, literature data are still conflicting about how obesity surgery can impact cancer development [3] since some evidence shows cancer risk reduction, while others show its increase.

In the United States, by 2030, 20,000 cases of esophageal cancer are predicted, of which approximately 75% will be classified as esophagus adenocarcinoma (EAC) [10].

This histologic type, used to represent only 10% of the cases worldwide, is gaining more notoriety nowadays because of its strict relationship with gastroesophageal reflux disease (GERD), a condition defined as the backflow of the gastric fluid through the esophagus [11].

Obese patients are a vulnerable group to esophagus cancer since they are nearly three times more likely to have GERD than the normal-weight population [12]. Being overweight leads to consequences in esophageal anatomy and function: impaired esophageal clearance, a higher incidence of hiatal hernias, and a higher intra-abdominal pressure [12].

All these changes contribute to GERD’s beginning and progression. The chronic injury of the esophageal mucosa caused by gastric acid fluids can damage the tissue integrity: the healthy stratified squamous epithelium is replaced by a columnar epithelium. When this metaplastic tissue hits at least 1 cm, it is named Barrett’s esophagus (BE), a well-known esophageal adenocarcinoma risk factor [13]. Sleeve gastrectomy emerged as a popular technique due to its simplicity and agility compared to Roux-en-Y-bypass.

This procedure can induce or worsen GERD because it causes modifications in the stomach’s anatomy by altering the angle of His and increasing intra-abdominal pressure [13]. A prospective study revealed that patients undergoing sleeve gastrectomy had almost a 50% chance of developing GERD after the surgery [14]. Also, cases of esophageal adenocarcinoma after SG had already been described in the literature [15].

However, bariatric surgery can also reduce cancer risk. A cohort of more than 300,000 patients performed in New York showed that, after obesity surgery, patients had a lower risk of developing female-specific cancers-breast, ovarian, and endometrial [16].

In the case of endometrial cancer, the procedure reduces by 71% the risk of uterine malignancy overall. This rate can reach 81% when the average weight is maintained after the surgery [17].

The attenuation of metabolic syndrome is the primary mechanism to explain the reduction. After weight loss, surgery promotes insulin resistance and inflammation reduces, improving estrogen regulation [8].

There are also some unclear aspects in the literature about the relationship between bariatric surgery and cancer risk. Some cohort studies reported conflicting results about the surgery’s impact on colorectal cancer (CRC) another malignancy with higher incidence among the obese population.

A multicentric Nordic study observed that obese patients who underwent bariatric surgery had an increased risk of developing colon cancer when compared to non-operated patients, especially after 10 or more years since the procedure [18]. Another retrospective study compared the colonoscopy results of obese patients before and after Roux-en-Y bypass. It showed a higher prevalence of serrated polyps—a precursor of CRC- 5 years post-operation [19].

Some factors may explain these outcomes, like the expression of some inflammatory biomarkers and anatomical-functional rearrangements noticeable after obesity surgery. Trials have shown that postoperative patients had increased levels of proinflammatory genes, such as cyclooxygenase-1 and cyclooxygenase-2, related to CRC pathogenesis [20].

Also, according to Kant et al. [21], a higher proliferation of the epithelial rectal cells, associated with apoptosis reduction, was observed 03 years after RYGB. Bariatric surgery, mainly RYGB, alters the natural physiology of the gastrointestinal tract due to the high bile acid flow created after the bypass [21].

The exposure of the gut’s mucosa to bile acids causes transformations in enteric pH and microbiota [22], which can stimulate tumorigenesis [23]. However, these findings disagree with the results obtained by a meta-analysis conducted by Wilson et al. that revealed bariatric surgery to be associated with a significantly lower risk of CRC (p = 0.003) [21].

Faced with all these gaps and conflicts, this systematic review and meta-analysis aim to elucidate the relationship between bariatric surgery and cancer risk.

#### Review question

What is the impact of bariatric surgery on cancer risk?

## Objectives

This systematic review and meta-analysis protocol will seek to answer the following key questions:

### Primary aim

1. Does bariatric surgery in obese patients impact the risk of obesity-related cancer development?

### Secondary aim

1. Does bariatric surgery modify cancer mortality rates?
2. Does the surgery technique impact obesity-related cancer development?
3. There is an association between histological type of tumor and surgery?
4. What is the average time from cancer diagnosis after bariatric surgery?
5. Is there statistical significance between variables such as age and gender and the incidence of obesity-related cancers post-bariatric surgery?

## Materials and methods

This systematic review and meta-analysis will be reported according to the Preferred Reporting Items for Systematic Reviews and Meta-Analyses (PRISMA-P) guidelines [24]. Also, this review protocol was written based on the Cochrane Handbook for Systematic Reviews of Interventions [25]. This protocol is registered with the International Prospective Register of Systematic Reviews [CRD42023432079].

### Inclusion criteria

This systematic review will include observational studies (case-control and cohort studies) about patients who underwent bariatric surgery due to metabolic syndrome. Will be accepted in any language and any year. Publications without peer review will be excluded from this review.

### The PECOT strategy

P—Patients with obesity and eligible for bariatric surgery.
E—Primary bariatric surgery (gastric bypass, gastric banding, or sleeve gastrectomy).
C—Patients with obesity undergoing pharmacological treatment and lifestyle modifications.
O—Event rate of obesity-related cancers.
T—Observational studies (Cohort and case-control studies).

### Types of patients

Participants will be adult patients who underwent bariatric surgery and with a diagnosis of obesity-related cancers without a specific histologic type or anatomical site. There will be no other age or gender restrictions.

### Types of interventions

Comparison of the prevalence of obesity-related cancer between patients who underwent bariatric surgery and those who received a clinical treatment based on pharmacological therapeutic and lifestyle changes.

### Type of outcome measures

The primary outcome to be evaluated will be the event rate of obesity-related cancers by including relative risk and odds ratio or hazard ratio. There are no additional outcomes to be assessed.

### Patient and public involvement

This work is a systematic review protocol; the research will be performed by searching literature from databases without including individual patient data. Thus, the search questions will not involve patients.

In the same way, the outcome measurements during the design and implementation of the study, such as the dissemination of the results, will not include patient data.

### Search strategy

To obtain the observational studies of this systematic review and meta-analysis protocol, the search will be performed in these databases: PubMed, ScienceDirect, Embase, CINAHL, LILACS, CENTRAL, Web of Science, Scopus and Cochrane Library.

The following Medical Subject Heading (MESH) terms will apply in the databases above:

(Obesity* OR Obesity, Morbid OR Overweight OR Nutrition Disorders) AND (Bariatric Surgery OR Gastric Bypass OR Sleeve Gastrectomy) AND (Neoplasms* OR Malignancy OR Malignancies OR Tumors OR Cancer) AND (Observational studies OR Cohort studies OR Case-control studies OR Retrospective studies OR (incidence OR incidence studies OR incidence study OR incidences OR studies, incidence OR prevalence OR prevalence studies OR prevalence study OR hazard ratio OR cox proportional hazards models OR hazard model, proportional OR hazard models, proportional OR hazards model, proportional OR odds ratio OR odds ratios).

### Other sources

Other studies may be included, coming from the reference list of retrieved articles, due to the enlargement of the data available to be included in the study, with the aim of developing a systematic review and meta-analysis most large and comprehensive.

## Data collection and analysis

### Selection of studies

**Fig 2:**
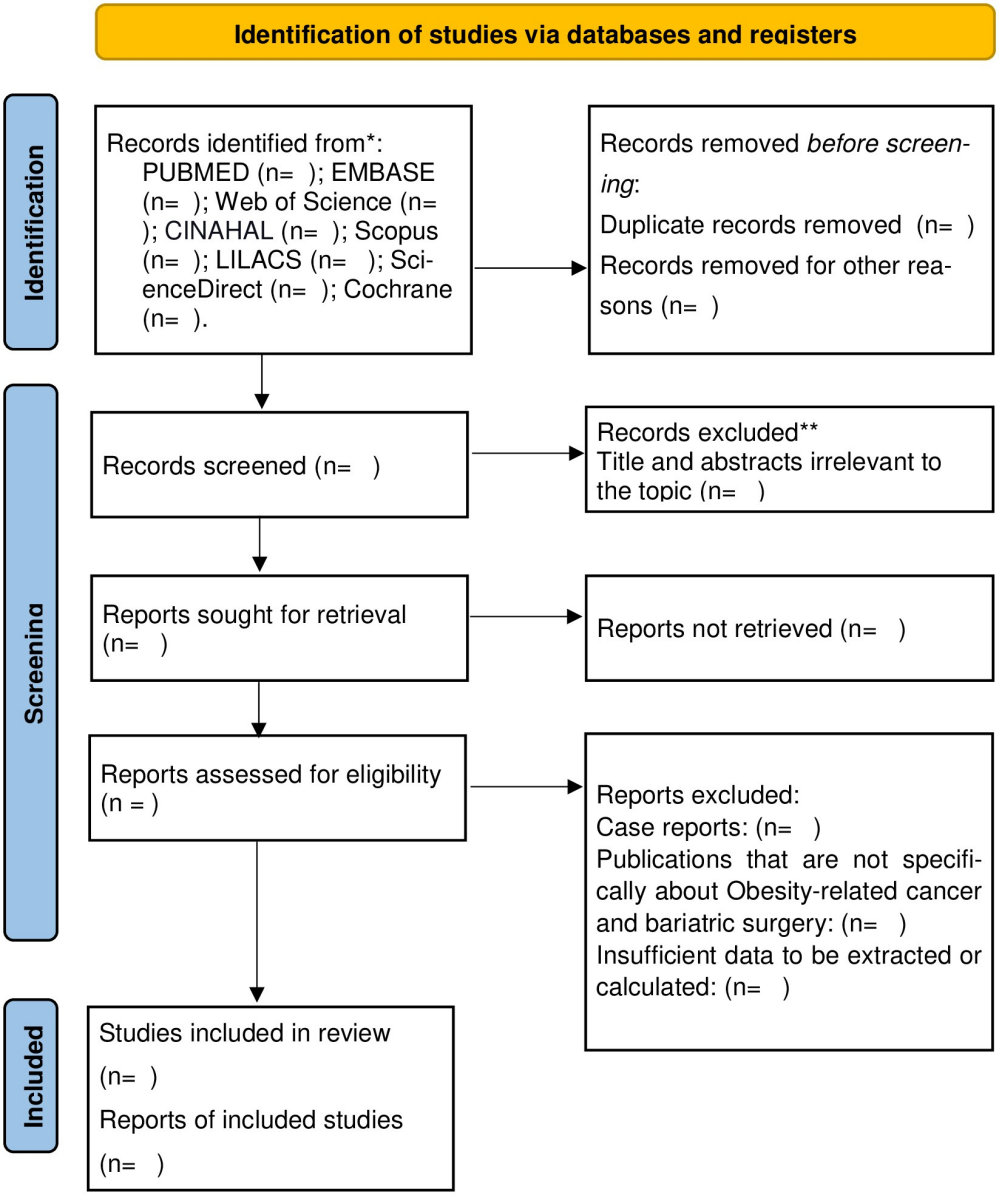
PRISMA flow diagram.

The researchers IAFF and CCSC used the Ryan Software to select the studies of interest, according to the inclusion criteria. After the independent reading of titles and abstracts, duplicate studies will be excluded. A third reviewer (AIA) will resolve the discrepancies. A PRISMA flow diagram (Fig 2) will be used to summarize the chosen articles.

### Data extraction and management

The researchers IAFF and CCSC will create and test a form of to extract the data from the observational studies. These researchers will resolve any discrepancies through discussion with the reviewer AIA.

The data extracted will include information on authors, year of publication, type of study, number of cases and controls, mean of age, gender, country, comorbidities, smoking status, alcohol consumption, type of neoplasms, anatomical region of malignancy, histological type (due to the possible influence in the carcinogenic pathway), weight at diagnosis, distant metastasis, type of bariatric surgery performed and follow-up.

### Addressing missing data

The authors will resolve any missing data through contact to authors or co-authors of the article. If not enough information is received, the data will be excluded from our analysis and the reasons will be explained in the discussion section.

### Risk of bias assessment

Two authors (BSC and AIA) will assess the risk of bias in each eligible study, independently, using the ROBINS-I tool [26] to dichotomous outcomes. Preference will be evaluated with three categories of judgment (unclear, low, or high) for individual elements.

A high score indicates a low risk of bias. Authors will be contacted if there is insufficient information to determine the precise risk of bias. Raters will resolve disagreements through a consensus with a third reviewer; if an agreement is unreachable, a fourth rater will arbitrate the case.

### Assessment of heterogeneity

The evaluation of heterogeneity will be estimated using I^2^. If the I^2^ value is under 50%, the heterogeneity is low, and a fixed-effect model will be used in the analysis. Otherwise, the heterogeneity will be considered high if the I^2^ value is 50% or more. They are using random effects models, along with the Mantel-Haenszel method assessment of the impact of heterogeneity on the selected studies.

### Analysis

Review Manager statistical program V.5.2.3 will be utilized to evaluate the data. A random-effects model for meta-analysis will be used because it is anticipated that there will be some degree of heterogeneity among the studies.

For dichotomous data, effect sizes will be expressed as risk ratios, odds ratios, or prevalence ratios; for continuous data, they will be defined as weighted mean differences with their respective 95% confidence intervals (CI).

Case-control estimates will be provided as odds ratios with 95% CI, whereas cohort estimates will be reported as risk or prevalence ratios with 95% CI. Case-control estimates will be provided as odds ratios with 95% CI, whereas cohort estimates will be notified as risk or prevalence ratios with 95% CI. I^2^ squared statistics will be used to gauge the level of statistical heterogeneity.

We will offer a narrative summary of the research findings when statistical pooling is not feasible, and there is significant heterogeneity. A sensitivity analysis will be carried out to investigate the effect of the included studies’ quality. If more than ten papers were included, publication bias would be evaluated using a funnel plot. Egger’s test (for continuous outcomes) will be used to assess funnel plot asymmetry.

### Grading quality of evidence

We will use the Grading of Recommendation Assessment, Development, and Evaluation (GRADE) [27] approach to grade the strength of evidence from the included data. The assessment summary will be incorporated into broader measurements to ensure the judgment of the risk of bias, consistency, directness, and precision.

These elements will supply the quality of the evidence. GRADE tool classifies the studies as low, moderate, and high quality. In case of insufficient information to quality-assess the studies, the authors will be contacted via e-mail. Two authors will independently evaluate this, and disagreements will be decided through discussion (third author).

#### Ethics and dissemination

This study will review the published data; thus, it is not necessary to obtain ethical approval. The findings of this systematic review will be published in a peer-reviewed journal.

## Discussion

Obesity is a growing public health problem worldwide, being present in almost all age groups. The World Health Organization defines obesity as a Body Mass Index (BMI) ≥ 30 kg/m^2^, while a BMI between 25 and 29.9 kg/m^2^ determines overweight [28].

In 2015, it was estimated that 605 million adults were obese, a prevalence respectively 6 and 2 times higher compared to the same age group in 1970 [29]. Obesity is infamous for its correlation with metabolic syndrome, type 2 diabetes, neoplasms, cardiovascular diseases, and the increased inflammatory process.

The excess visceral adiposity, typical of obesity, is responsible for increasing the number of molecules that induce cancer pathogenesis, such as leptin, resistin, and other adipokines, in addition to pro-inflammatory cytokines such as IL-1, IL-6, IL -8, and TNF-α, elevating the risk of neoplasms [30].

Several studies have sought to establish an association between being overweight and a higher risk of developing cancer. In 2016, the International Agency for Research on Cancer (IARC) published a study revealing that obese patients had a higher risk of developing cancers such as gastric cardia, liver, gallbladder, pancreas, kidneys, ovary, thyroid, multiple myeloma, and meningioma, in addition to esophageal adenocarcinoma [5].

Treating obesity and metabolic syndrome involves lifestyle and behavior changes, pharmacological therapies, and, when correctly indicated, surgical intervention.

Due to the increasing number of bariatric surgeries, recent studies have sought to understand whether or not such procedures influence the risk of developing cancer. Most studies point to a reduction in the risk of cancer after bariatric surgery; however, this association seems uncertain for certain types of cancer.

A retrospective observational study evaluated the development of esophageal and gastric cancer in 170 patients in 75 centers from 25 countries who underwent bariatric surgery between 1985 and 2020.

The study shows that almost one-third of patients presented with metastatic disease, and 85% of the tumors were adenocarcinoma [31]. In contrast, another recent cohort with more than 1 million patients in France showed a similar risk of developing colorectal cancer (CRC) among individuals with morbid obesity and the general population aged between 50 and 75 years [32].

However, among individuals with morbid obesity, those who did not undergo surgery had a 34% greater risk of having CRC than those who underwent bariatric surgery [32]. In addition, studies show an increased risk of developing certain types of cancer after surgical treatment for weight loss, such as colorectal cancer [33–35].

The reasons for the variability of the results found involve the age and gender of the patients and the notorious hormonal influence, the designs of the evaluated studies (such as Systematic review or observational studies), and the different surgical procedures performed.

Due to the infeasibility of carrying out randomized clinical trials to evaluate the influence of surgical procedures for treatment of obesity in the development of cancer, it is justified to develop this systematic review and meta-analysis, with the aim of a better elucidation of the association of bariatric surgery in the process of carcinogenesis, as well as to evaluate the relationship between the different types of bariatric procedures (such as sleeve gastrectomy and Roux-en-Y Gastric bypass) and their influence on the development of cancer.

## Supporting information

S1 Checklist PRISMA-P 2015 checklist. (DOCX)
